# Preliminary Evidence That Yoga Practice Progressively Improves Mood and Decreases Stress in a Sample of UK Prisoners

**DOI:** 10.1155/2015/819183

**Published:** 2015-07-30

**Authors:** Amy C. Bilderbeck, Inti A. Brazil, Miguel Farias

**Affiliations:** ^1^Department of Psychiatry, Oxford University, Oxford OX3 7JX, UK; ^2^Donders Institute for Brain, Cognition and Behaviour, 6525 EZ Nijmegen, Netherlands; ^3^Pompestichting, Nijmegen, 6532 CN Nijmegen, Netherlands; ^4^Research Centre for Psychology, Behaviour and Achievement, Coventry University, Coventry CV1 5FB, UK

## Abstract

*Objectives*. In the first randomized controlled trial of yoga on UK prisoners, we previously showed that yoga practice was associated with improved mental wellbeing and cognition. Here, we aimed to assess how class attendance, self-practice, and demographic factors were related to outcome amongst prisoners enrolled in the 10-week yoga intervention.* Methods*. The data of 55 participants (52 male, 3 female) who completed a 10-week yoga course were analysed. Changes in pre- and postyoga measures of affect, perceived stress, and psychological symptoms were entered into linear regression analyses with bias-corrected and accelerated bootstrap confidence intervals. Class attendance, self-practice, demographic variables, and baseline psychometric variables were included as regressors.* Results*. Participants who attended more yoga classes and those who engaged in frequent (5 times or more) self-practice reported significantly greater decreases in perceived stress. Decreases in negative affect were also significantly related to high frequency self-practice and greater class attendance at a near-significant level. Age was positively correlated with yoga class attendance, and higher levels of education were associated with greater decreases in negative affect.* Conclusions*. Our results suggest that there may be progressive beneficial effects of yoga within prison populations and point to subpopulations who may benefit the most from this practice.

## 1. Introduction

There is emerging evidence that the practice of yoga may play a significant role in prison rehabilitation programmes [1, 2]. In community and clinical samples, yoga has been associated with numerous physical [3, 4] and psychological benefits, including improved mood and wellbeing, reduced depression, anxiety and perceived stress, and reduced aggression [5–9]. Based on these findings it can be expected that yoga has the potential to have a significantly positive impact on the mental health of prison populations, particularly as offenders display increased stress reactivity and extreme forms of aggression [10, 11]. Indeed, there is some limited but promising evidence that yoga can ameliorate symptoms of depression and anxiety in offenders [2], as well as helping improve sleep, mood, and social behaviour [12]. Adding to this body of research, we recently showed in a randomized trial that participation in a 10-week yoga and meditation course was associated with improved mood, decreased stress, and reduced indices of psychological distress among UK prisoners when compared with a control group of prisoners [1]. Improved performance on a cognitive task was also observed among prisoners who took part in the yoga intervention compared to control participants.

Previously, we looked at the effects of yoga practice by comparing the pre- and postintervention scores between the yoga and control groups; here, our aim was to investigate what might underpin the beneficial effects of yoga within this prison population.

To do this, we tested whether effects were driven by the level of attendance of classes, so that a greater attendance would be associated with better mental health and cognitive outcomes. Adherence to treatment regimens is often associated with outcome; a relationship which has been reported as stronger for nonmedication compared to medication-based interventions [13]. When class attendance is low, the “dose” of the intervention may be considered reduced, and the intervention may fail to deliver full benefits. As the “dose” in complex interventions may also be dependent on engagement outside of structured classes, we also tested whether frequency of self-practice had an influence on participants' mood, levels of stress, and psychological distress.

In addition, we examined whether factors such as age, education, ethnicity, and current relationship status were predictive of outcome following the yoga course. We also included gender as a demographic regressor in our model but were cautious when interpreting the results as data were only available for 3 women. Inclusion of these variables in our model was primarily exploratory, in order to identify potential prisoner subpopulations who might benefit especially from rehabilitation programs involving yoga. Inclusion of these variables also allowed us to statistically account for their potential influence on outcomes: for example, relationship status has been found to have an impact on wellbeing [14, 15], including within prison populations [16].

## 2. Methods

### 2.1. Participants

In our original study design [1], 167 prisoners (155 male, 12 female) were randomly assigned to the yoga or control group. The present analysis, which only describes the data from those participants who completed the 10-week yoga course, included 87 prisoners (81 male, 6 female) from 7 Category B or C West Midland prisons. Participating prisons were Her Majesty's Prison (HMP) Dovegate; HMP Hewell; HMP Featherstone; HMP Stafford; HMP Shrewsbury; and HMP Young Offenders' Institution (YOI) Swinfen Hall and one women's prison HMP YOI Drake Hall.

The study was approved by Ethics Committees of the British National Health Services and the Ministry of Justice, and all participants provided written informed consent to take part. Prisoners with a major medical condition and those actively being treated for psychiatric illness or substance abuse were excluded from taking part in the study. Sixty-one of the 87 prisoners randomly allocated to the “yoga” group attended two assessment sessions: the first assessment session was conducted one week before the start of the yoga intervention (henceforth “T1”) and the second took place one week after completion of the 10-week course (henceforth “T2”). Chi-square testing showed that participants who attended both assessment sessions did not differ from those who attended only the first session, in terms of educational qualifications achieved (*χ*(2) = 0.446, inside the 95% CI of [0.00,7.38], *p* = 0.45), ethnicity (*χ*(1) = 0.001, inside the 95% CI of [0.00,3.84], *p* = 0.98), and current relationship status (*χ*(1) = 0.215, inside the 95% CI of [0.00,5.02], *p* = 0.64). There was a tendency for participants who attended both assessment sessions to be older than those who only attended the first session, *F*(1,85) = 3.78, CI [−9.91,0.11], *p* = 0.055. See Table 1 for demographic data of participants who did and did not complete both assessment sessions.

For the 61 participants who completed both sessions, 58 (95%) were male; age ranged from 20 to 66 years with a mean of 36.74 (s.d. 11.45); and 46 (75%) were Caucasian (of those remaining, 8 were black, 3 were Asian, and 3 were of mixed race).

### 2.2. Procedure

The yoga course consisted of a total of 10 classes, once per week over ten weeks, and each class had a two-hour duration. Classes consisted of a standardized set of hatha yoga postures and stretches; example poses can be seen in Bilderbeck et al. [1]. Roughly the first ten minutes of each class was spent doing postures and movements to warm the body, and this was followed by postures involving larger movements and deeper stretches of the limbs and spine. Each class ended with a period of relaxation and meditation, consisting first of breathing exercises (either alternate nostril breathing (*nadi shodan*) or “skull shining breath” (*kapalbhati*) or both, duration roughly 5 minutes), followed by lying in “corpse pose” (*savasana*; duration roughly 5 minutes), followed by a final seated meditation with attention directed to the breath (see supplementary material for meditation instructions provided to participants; duration 10 minutes, building towards 20 minutes for the final 5 weeks of classes (see Supplementary Material available online at http://dx.doi.org/10.1155/2015/819183)).

At T1 (one week before the start of the intervention), we collected sociodemographic information such as gender, age, level of education, ethnicity, and relationship status. Participants also completed the Positive and Negative Affect Scale (PANAS; [17]) as a measure of affect, the Perceived Stress Scale (PSS; [18]) as a measure of recent stress, and the Brief Symptom Inventory (the BSI; [19]) as a measure of psychological distress. At T2 (one week after intervention) participants were assessed using the same measures (cf. [1]). Participants were asked at the T2 assessment to report the average frequency that they practised yoga in their own time, in addition to the weekly classes. This self-practice data was recorded in the following categories: never; once or twice per week; 3 or 4 times per week; 5 times or more per week. Participants were asked to respond to PANAS, PSS, and BSI items whilst considering the time period encompassing the previous week.

### 2.3. Statistical Analysis

We conducted a series of multiple linear regression analyses to determine whether the number of yoga classes attended contributed to explaining the variance in psychological outcomes, after statistically controlling for demographic variables and key baseline psychometrics. We used bootstrapping to conduct all multiple linear regression analyses and obtain confidence intervals, as an alternative to inferences based on parametric assumptions [20]. All analyses were conducted using SPSS version 22.0 [21].

We created models in which our outcome measures were self-reported change (T2-T1; annotated with the symbol “Δ”) in (i) perceived stress, (ii) psychological distress, (iii) positive affect, and (iv) negative affect. Independent variables comprised (i) number of yoga classes attended; (ii) age; (iii) relationship status (categorized as single or in a relationship); (iv) ethnicity (Caucasian or non-Caucasian); (v) education (classified as no academic qualifications; GCSEs attained; or higher education degree attained); (vi) baseline (T1) perceived stress, (vii) baseline psychological distress, (viii) baseline positive affect, and (ix) baseline negative affect. The latter 4 baseline psychometric variables were included for robustness as variables of no interest; we note that, due to regression-to-the-mean trends within data sets such as this [22] and the lack of a control group in this analysis, any relationship specifically between baseline psychometrics and key dependent variables cannot not be easily interpreted (see also Table 2 legend). We also included in the model regressors to capture the effects of frequency of self-practice. This data had been collected as a self-report ordinal variable with 4 levels (“never”; “1-2 times per week,” “3-4 times per week,” and “5 or more times per week”). We recoded this data into 3 binary “dummy” variables, with no self-practise as the reference.

The number of samples was set to 5,000. Beta-values in the results below are presented together with the (±) bootstrapped standard error, the corresponding 95% confidence interval (CI), and bootstrap-based *p* values.

## 3. Results

Six participants were excluded from the analysis; 4 did not attend any yoga classes and there was no record of number of classes attended for 2 other participants. This left a total of 55 participants for whom data was analysed. Internal reliability was acceptable for all questionnaire measures for Time 1 and Time 2 (Cronbach's *α* ranging from 0.80 to 0.96).

We first explored the variables associated with attendance of the yoga classes. Attendance and age were positively related, *β* = 0.074 ± 0.031, CI [0.011, 0.134], *p* = 0.023. No demographic variables (neither ethnicity, relationship status, nor educational qualification achieved) were associated with yoga class attendance (*p* values = 0.11–0.95).

In terms of levels of self-practice, 21 participants (38.1%) reported doing no extra practise outside of the weekly classes; 8 (14.5%) reported doing self-practice sessions once or twice per week; 13 (23.6%) reported self-practice 3 or 4 times per week, and 12 (21.8%) participants reported self-practice 5 times or more per week (data was not reported for one participant). Interestingly, mean class attendance did not differ as a function of reported frequency of self-practice (−0.02 < *β*s < 0.003, *p*s > 0.35).

**Figure 1:**
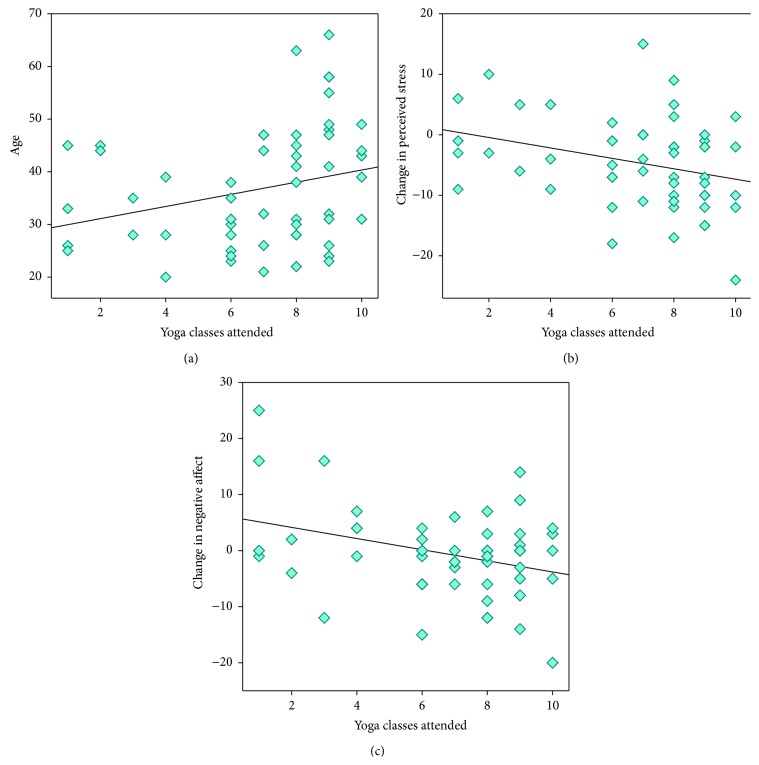
Scatter-plot and regression lines for yoga classes attended plotted as a function of (a) age; (b) change (T2-T1) in perceived stress; and (c) change in negative affect, for 55 prisoners enrolled in a 10-week yoga course.

**Figure 2:**
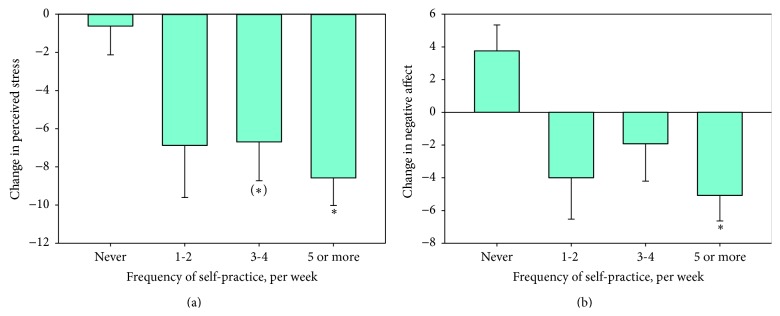
Bar chart of change in perceived stress (T2-T1; (a)) and change in negative affect (b) as a function of weekly frequency of self-practice, for 55 prisoners enrolled in a 10-week yoga course. ^*∗*^Significant difference compared to the reference of no self-practice at *p* < 0.05. (*∗*) Near-significant difference compared to the reference of no self-practice at *p* < 0.1.

We then examined the factors which were related to greater change in the psychometric variables (see Table 2). Greater improvement (i.e., decrease) in perceived stress was significantly related to greater yoga class attendance (Figure 1). Self-practice 5 or more times per week was significantly associated with decreased perceived stress, as was self-practice 3-4 times per week at a near-significant level, in both cases relative to no self-practice (Figure 2). Greater reduction in negative affect was also significantly associated with self-practice 5 or more times per week. Greater decreases in negative affect were also associated with greater class attendance, albeit at trend level (Figure 1). Change in negative affect was additionally related to level of education. Namely, greater decreases in negative affect were associated with higher levels of education. These effects held after the considerable influence of baseline psychometrics had been statistically accounted for (see Table 2 and also Supplementary Table 1).

Changes in psychological distress and positive affect were unrelated to yoga class attendance or frequency of self-practice.

## 4. Discussion

Participation in a larger number of yoga classes was associated with psychological benefits among prisoners. Participating in more yoga classes was related to significantly greater decreases in perceived stress and greater reductions in negative affect at a near-significant level. Higher levels of self-practice (5 times or more per week) were also related to reduced perceived stress and negative affect. These effects were robust when statistically controlling for the potential (and demonstrated) effects of demographic variables including age and academic qualifications, as well as the participants' baseline psychometrics. These data extend our previous findings [1] by suggesting that yoga may have progressive influences on mood and stress, with greater improvements as prisoners engage in routine practice, in such a way that may potentially extend beyond the 10-week intervention. The observed relationships between self-practice and outcomes imply that benefits are more pronounced when engagement in self-practice is high (5 or more times per week); however our small sample size makes it possible that less frequent self-practice is associated with benefits which we were not powered to detect in these analyses. Overall and based on these observations we suggest that, by improving prisoners' mental health and addressing known criminogenic agents including negative affective states [23], yoga may aid rehabilitative programs by enhancing emotional regulation and potentially facilitating greater behavioural control.

In our original analysis, we observed that participants who completed the yoga course demonstrated a significant increase in positive affect compared to their control group counterparts. Yoga practice did not modify negative affect. This represents an apparent inconsistency with the present results, where increased yoga class attendance and self-practice were associated with greater decrease in negative affect, but with no effect on positive affect. However, these two results are not mutually exclusive; we take this combination of findings to point to more subtle effects of the yoga course on affect, which may be observed (or not) depending on the specific methodological and statistical approaches employed. This may in part account for inconsistencies observed in the field of yoga and meditation research more broadly, where effects of yoga on both positive and negative affect are sometimes, but not always, observed [24–27]. We note that negative affect did, in fact, decrease amongst participants who completed the yoga programme, but that this did not reach a statistically significant level compared to the control group [1]; similarly other randomized controlled trials of yoga have reported within-group differences in affect that did not reach significance in between-group contrasts [25].

We found that older participants were more likely to adhere to the yoga course. It has been previously noted that, within similar age ranges to those observed in the present study, older participants are more likely to adhere to diet and exercise interventions [28–30]. Compliance with antidepressant treatment amongst depressed prisoners has also been positively related to age [31]. Older prisoners may be more open to engaging in yoga or demonstrate greater compliance with a new regime and therefore potentially more likely to experience benefits from rehabilitation programs involving yoga. Our data may indicate that there is a need to provide greater support for younger incarcerated individuals to adhere to interventions, including those that are complex and longer term.

We also observed that those individuals who had achieved higher levels of educational qualifications showed greater decreases in negative affect following the yoga intervention. This highlights how certain populations may benefit especially from such interventions. In accounting for this finding, we note that, in the general population, higher levels of education are associated with more frequent use of complementary and alternative therapies, including yoga [32]. Although none of our participants had any previous experience of practising yoga themselves, those with higher levels of educational attainment may have found the concepts and practices of yoga more accessible, leading to potentially more engagement with this form of intervention and greater associated benefits.

There are a number of important caveats to consider when interpreting the positive association between yoga practice and adherence. Personality factors, including conscientiousness and social desirability, may motivate both greater class attendance/self-practice as well as reports of improved mood. As we did not measure these factors we were unable to test these potential interactive or mediating effects on outcomes of the yoga intervention. Furthermore, it has been suggested that adherence to treatment, independently of treatment itself, is associated with improved outcomes [33, 34]. Adherence may be a marker for broader engagement in health-promoting behaviours; higher self-efficacy; or personality traits related to motivation or goal-directed behaviour. However, links between adherence and outcomes in more complex mind-body interventions such as those involving yoga have not been consistently observed [35, 36]. Exploring these links will require further investigation utilizing, for example, experimental designs that include adherence tracking within both control and intervention groups. A further important caveat involves the direction of causality in our key findings. It is possible that prisoners who did not experience improvement in affect and wellbeing during the 10-week yoga course attended fewer sessions, rather than reduced attendance resulting in lessened benefits of the course. Roughly 30% of participants dropped out of the study, perhaps raising questions about generalizability of our findings. However, this seemingly high drop-rate is quite common in intervention studies that include offender populations [37]. Finally, investigation is needed into the efficacy of yoga practice in more clearly defined criminological samples in which participants are selected based on criminal history and, if possible, also psychiatric wellbeing.

Our findings provide support for the idea that yoga is effective in improving psychological wellbeing among inmates and may form a part of effective rehabilitation programmes. These data emphasize the potentially enhanced benefits of sustained, regular practise, and point to the potential therapeutic value of yoga in other nonforensic populations with high psychiatric morbidity. However, the need for further research is also clear. Our sample size was small, and it will be important to replicate these findings in a larger sample. It is uncertain whether the key benefits of yoga interventions are specifically linked to elements of the yoga practice (poses; breathing techniques; meditation) or broader elements of these forms of complex intervention (effects of physical exercise; social interaction with a teacher or with other practitioners). Potential overlaps with the mechanisms of action of more extensively researched practices such as mindfulness meditation are also currently poorly understood. We were unable to collect data on penitentiary variables (e.g., length of sentence, nature of crime) and were therefore unable to control for or test the potential influence of these factors. Finally there is, as yet, no standardized or widely accepted programme of yoga that might focus research efforts. Nevertheless, these data add to a body of research suggesting that the benefits of yoga in forensic and clinical populations merit further exploration.

## Supplementary Material

The Supplementary Material contain information about the instructions that were provided to participants during the final seated meditation, together with the full results of analyses exploring effects of attendance, self-practice, demographic variables, and baseline psychometrics against outcome .

## Figures and Tables

**Table 1:** Key demographic variables for those participants who did (*N* = 61) and those who did not (*N* = 26) complete the study, that is, those who completed assessment sessions at Time 1 (T1) and Time 2 (T2), compared to those who “dropped out” after T1.

	Completed only T1 (*N* = 26)	Completed T1 and T2 (*N* = 61)
Age (mean ± S.E.)	31.35 ± 1.90	36.25 ± 1.43
Gender (M)	23 (88%)	58 (95%)
Relationship status		
Single	20 (77%)	44 (72%)
Married or has partner	6 (23%)	17 (28%)
Ethnicity		
Caucasian	20 (77%)	46 (75%)
Non-Caucasian	6 (23%)	14 (25%)
Educational qualifications achieved		
None	10 (38%)	20 (33%)
O-levels/GCSEs	10 (38%)	23 (38%)
A levels or higher	6 (23%)	18 (29%)

**Table 2:** Summary of key statistical results.

	*β* in final model	95% CI	*p* value
Δ *perceived stress *			
Yoga classes attended	−1.053 ± 0.383	[−1.850, −0.341]	*p* = 0.010^*∗*^
Self-practice 3-4 times per week^†^	−4.650 ± 2.293	[−9.114, 0.064]	*p* = 0.052^(*∗*)^
Self-practice 5 or more times per week^†^	−5.329 ± 2.408	[−10.147, −0.636]	*p* = 0.032^*∗*^
Psychological distress at T1	−0.450 ± 0.169	[−0.777, −0.109]	*p* = 0.013^*∗*^
Δ *psychological distress *			
Perceived stress at T1	−0.584 ± 0.253	[−1.176, −0.233]	*p* = 0.017^*∗*‡^
Δ *positive affect *			
Positive affect at T1	−0.609 ± 0.139	[−0.874, −0.33]	*p* = 0.001^*∗*‡^
Δ *negative affect *			
Yoga classes attended	−0.914 ± 0.473	[−1.666, 0.217]	*p* = 0.074^(*∗*)^
Self-practice 5 or more times per week^†^	−4.863 ± 2.187	[−9.128, −0.441]	*p* = 0.033^*∗*^
Gender	9.331 ± 3.672	[1.945, 16.639]	*p* = 0.018^*∗*^
Educational qualifications achieved	−2.913 ± 1.042	[−4.947, −0.825]	*p* = 0.019^*∗*^
Negative affect at T1	−0.563 ± 0.186	[−0.931, −0.187]	*p* = 0.006^*∗*‡^

First column shows, in italics, dependent values for each regression model. Beta-values (±bootstrapped standard errors) are shown, together with 95% confidence intervals (CIs) and bootstrapped *p* values. Only regressors significant at ^*∗*^ *p* < 0.05 and ^(*∗*)^ *p* < 0.1 are presented here (see Supplemental Table 1 for full results of regression analyses). ^†^Self-practice regressors are coded against a reference of no yoga self-practise. ^‡^Note that, due to regression-to-the-mean phenomena, any relationship specifically between baseline psychometrics and key dependent variables should be interpreted with care (see also main text).
