# Supplementary material for: Preliminary Evidence That Yoga Practice Progressively Improves Mood and Decreases Stress in a Sample of UK Prisoners
Source: Evid Based Complement Alternat Med. 2015 Jul 30;2015:819183. doi: 10.1155/2015/819183 (PMC4534616; doi:10.1155/2015/819183)
Supplement: Supplementary file 1 — The Supplementary Material contain information about the instructions that were provided to participants during the final seated meditation, together with the full results of analyses exploring effects of attendance, self-practice, demographic variables, and baseline psychometrics against outcome . [file 819183.f1.docx]

**Instructions provided to participants during final seated meditation**

Yoga teachers were provided with three techniques for focussing attention during meditation, with instructions as below. Yoga teachers were asked to select a technique for each given class or individual, as they felt was appropriate.

1. As you breathe in, count silently in your mind “1.” As you breathe out, count “2.” Breathe in and count “3.” Breathe out and count “4.” Keep going to “10” and then begin start again at “1.” If you lose count, simply come back to “1.”

2. As you breathe in, simply pay attention to the breath. As you breathe out, count “1.” Breathe in, pay attention to the breath. Breathe out, count “2.” Keep going to “10” and start again at “1.”

3. As you breathe in and out, simply pay attention fully to the breath, with each inhalation and each exhalation.

**Supplementary Table 1 (below).** Full results of regression analyses exploring effects of attendance, self-practice, demographic variables, and baseline psychometrics against outcome for 55 participants who completed the 10 week yoga course.

|  | **β in final model** | **95% CI** | ***p-value*** |
| --- | --- | --- | --- |
| ***Δ Perceived Stress*** |  |  |  |
| Yoga classes attended | -1.053±0.383 | [-1.850, -0.341] | p = 0.010 |
| Self-practice 1-2 times per week | -3.661±2.911 | [-9.849, 1.672] | p = 0.208 |
| Self-practise 3-4 times per week | -4.650±2.293 | [-9.114, 0.064] | p = 0.052 |
| Self-practice 5 or more times per week | -5.329±2.408 | [-10.147, -0.636] | p = 0.032 |
| Gender | 2.229±3.625 | [-4.672, 9.662] | p = 0.521 |
| Age | -0.030±0.094 | [-0.204, 0.172] | p = 0.737 |
| Ethnicity | 1.748±2.197 | [-2.783, 5.979] | p = 0.420 |
| Relationship Status | -1.326±2.718 | [-6.518, 4.294] | p = 0.619 |
| Educational qualifications achieved | 0.709±1.192 | [-1.621, 3.078] | p = 0.533 |
| Psychological Distress at T1 | -0.450±0.169 | [-0.777, -0.109] | p = 0.013 |
| Perceived Stress at T1 | -0.021±0.043 | [-0.126, 0.052] | p = 0.546 |
| Positive Affect at T1 | 0.019±0.152 | [-0.288, 0.303] | p = 0.907 |
| Negative Affect at T1 | 0.091±0.256 | [-0.372, 0.631] | p = 0.704 |
| ***Δ Psychological Distress*** |  |  |  |
| Yoga classes attended | -2.015±1.558 | [-4.963, 1.304] | p = 0.205 |
| Self-practice 1-2 times per week | -17.872±11.137 | [-41.696, 0.94] | p = 0.129 |
| Self-practise 3-4 times per week | -11.102±8.689 | [-27.481, 7.158] | p = 0.220 |
| Self-practice 5 or more times per week | -3.949±10.62 | [-24.39, 17.203] | p = 0.710 |
| Gender | 13.454±16.29 | [-19.489, 45.369] | p = 0.400 |
| Age | -0.195±0.291 | [-0.827, 0.313] | p = 0.488 |
| Ethnicity | 14.498±9.298 | [-3.247, 33.018] | p = 0.153 |
| Relationship Status | 1.474±10.099 | [-17.158, 22.231] | p = 0.888 |
| Educational qualifications achieved | -1.944±4.247 | [-10.294, 6.576] | p = 0.630 |
| Psychological Distress at T1 | 0.069±0.752 | [-1.464, 1.505] | p = 0.920 |
| Perceived Stress at T1 | -0.584±0.253 | [-1.176, -0.233] | p = 0.017 |
| Positive Affect at T1 | 0.061±0.634 | [-1.284, 1.207] | p = 0.923 |
| Negative Affect at T1 | 0.311±0.988 | [-1.57, 2.366] | p = 0.752 |
| ***Δ Positive affect*** |  |  |  |
| Yoga classes attended | 0.683±0.541 | [-0.204, 1.948] | p = 0.178 |
| Self-practice 1-2 times per week | -4.793±3.516 | [-12.126, 1.795] | p = 0.181 |
| Self-practise 3-4 times per week | -1.790±3.257 | [-8.307, 4.31] | p = 0.586 |
| Self-practice 5 or more times per week | -3.810±3.293 | [-10.517, 2.408] | p = 0.252 |
| Gender | 1.220±5.47 | [-8.454, 13.03] | p = 0.816 |
| Age | 0.088±0.124 | [-0.144, 0.338] | p = 0.485 |
| Ethnicity | -2.001±2.77 | [-7.321, 3.623] | p = 0.477 |
| Relationship Status | 0.636±2.685 | [-4.957, 5.7] | p = 0.813 |
| Educational qualifications achieved | 0.204±1.462 | [-2.813, 2.972] | p = 0.889 |
| Psychological Distress at T1 | -0.386±0.233 | [-0.871, 0.037] | p = 0.101 |
| Perceived Stress at T1 | 0.045±0.048 | [-0.042, 0.154] | p = 0.269 |
| Positive Affect at T1 | -0.609±0.139 | [-0.874, -0.33] | p = 0.001 |
| Negative Affect at T1 | 0.0541±0.246 | [-0.462, 0.51] | p = 0.820 |
| ***Δ Negative affect*** |  |  |  |
| Yoga classes attended | -0.914±0.473 | [-1.666, 0.217] | p = 0.074 |
| Self-practice 1-2 times per week | -1.579±2.488 | [-6.937, 2.97] | p = 0.520 |
| Self-practise 3-4 times per week | -1.990±2.336 | [-6.011, 2.992] | p = 0.418 |
| Self-practice 5 or more times per week | -4.863±2.187 | [-9.128, -0.441] | p = 0.033 |
| Gender | 9.331±3.672 | [1.945, 16.639] | p = 0.018 |
| Age | 0.019±0.082 | [-0.144, 0.174] | p = 0.814 |
| Ethnicity | -1.676±1.997 | [-5.666, 2.195] | p = 0.404 |
| Relationship Status | 3.007±2.18 | [-1.508, 7.092] | p = 0.189 |
| Educational qualifications achieved | -2.913±1.042 | [-4.947, -0.825] | p = 0.019 |
| Psychological Distress at T1 | 0.266±0.17 | [-0.07, 0.607] | p = 0.126 |
| Perceived Stress at T1 | -0.056±0.048 | [-0.17, 0.033] | p = 0.185 |
| Positive Affect at T1 | 0.077±0.105 | [-0.123, 0.288] | p = 0.472 |
| Negative Affect at T1 | -0.563±0.186 | [-0.931, -0.187] | p = 0.006 |
